# A Genome-Wide Analysis of the *BAM* Gene Family and Identification of the Cold-Responsive Genes in Pomegranate (*Punica granatum* L.)

**DOI:** 10.3390/plants13101321

**Published:** 2024-05-10

**Authors:** Longbo Liu, Suwan Xu, Lehao Zhang, Jie Zheng

**Affiliations:** School of Life Science, Huaibei Normal University, Huaibei 235000, China; liulb@chnu.edu.cn (L.L.); 20221504045@chnu.edu.cn (S.X.); 20211504052@chnu.edu.cn (L.Z.)

**Keywords:** pomegranate (*Punica granatum* L.), BAM gene family, *PgBAM4*, cold stress, transcriptome

## Abstract

Beta-amylases (BAMs, EC 3.2.1.2), belonging to a multigene family, play a pivotal role in starch breakdown and are also involved in hormonal and stress responses, notably to cold stress. Pomegranate trees (*Punica granatum* L.) are adapted to warm climates and are sensitive to cold temperatures. In this study, we analyzed eight *PgBAM* genes from the pomegranate genome dataset. These members unevenly distributed across chromosomes and were categorized into four groups based on their orthologous members. The motif composition was highly consistent among most members. In contrast, exon numbers and arrangements were conserved within groups or subgroups, whereas significant diversity was observed between different groups. A syntenic analysis revealed that three *PgBAM* members (*PgBAM1/4/5*) showed a total of 11 syntenic relationships with the *BAM* members from Arabidopsis, kiwifruit, and Chinese white pear, respectively. Promoter binding motif prediction suggested potential roles for *PgBAMs*’ genes in light, stress, hormones, and development signaling. Gene expression indicated that *PgBAM4* was predominantly expressed in leaves, *PgBAM7* in flowers, and *PgBAM8* in roots and leaves and during fruit ripening, particularly in pericarp development. A transcriptome analysis identified the starch and sucrose metabolism pathway (map00500) as a key factor in the cold stress response of cold-sensitive cultivar ‘Tunisia’ seedlings. *PgBAM4* exhibited remarkable expression and was closely associated with the cold-responsive *BAM* genes, characterized by a closer phylogenetic relationship, conserved catalytic residues, and similar secondary and tertiary structures. Moreover, the differences in soluble sugar levels and *PgBAM4* expression were closely associated with the varying cold stress resistance observed between ‘Tunisia’ and ‘Sanbai’ seedlings. Furthermore, yeast one-hybrid assays confirmed that PgCBF7, a critical transcription factor for enhancing freezing tolerance, binds to the promoter region of *PgBAM4*. Our findings provide a systematic overview of the *PgBAM* gene family and shed new light on the regulatory mechanisms underlying cold stress tolerance in pomegranate.

## 1. Introduction

Starch, a complex glucose polymer, is a primary product of photosynthesis carbon assimilation, synthesized in the chloroplast during daylight. Starch metabolism plays multiple roles in the growth and development of sink organs, as well as in mitigating the effects of abiotic stress [1]. The starch metabolism can be functionally classified into synthetic and degradative reactions, which are catalyzed by a variety of enzymes. β-Amylases (E.C. 3.2.1.2, BAMs) are vital enzymes that facilitate the degradation of starch into maltose and are involved in controlling plant growth, development, and response to abiotic stress [2,3].

BAMs belong to a multigene family, but not all members are active enzymes [4]. In *Arabidopsis thaliana*, the nine *BAM* family members can be categorized into two groups based on their enzymatic activity. AtBAM1-3 and AtBAM5-6 are enzymatically active, with four members (AtBAM1-3 and AtBAM6) specifically functioning in chloroplasts, only AtBAM5 being exclusively cytosolic [5]. AtBAM1 and AtBAM3 exhibit cell-type-specific functions. AtBAM3 is responsible for the primary BAM activity in leaf mesophyll starch metabolism, and the downregulation of *AtBAM3* results in a starch-excess phenotype [6]. Under cold stress, *AtBAM3* (*AtBMY8*) is specifically induced, and its knockdown leads to decreased levels of maltose, glucose, fructose, and sucrose, increasing the sensitivity of the photosynthetic electron transport chain to freezing stress [7]. Comparatively, *AtBAM1* is highly expressed in guard cells and plays a significant role in rapid breakdown of guard cell starch to facilitate stomata opening [8]. During osmotic stress, AtBAM1 is activated and contributes to the degradation of starch, which in turn leads to the accumulation of sugar and proline [3]. AtBAM5 may participate in the degradation of starch released from the plastids of the phloem sieve elements [4]. The function of AtBAM2 and AtBAM6 in starch degradation remains unknown [5]. The other four members, AtBAM4 and AtBAM7-9, are catalytically inactive. Despite its lack of enzymatic activity, AtBAM4 is crucial for the regulation of leaf starch degradation. Mutants deficient in *AtBAM4* show a starch-excess phenotype [4]. AtBAM9 shares a close phylogenetic relationship with AtBAM4; however, its sequence exhibits a higher degree of conservation among diverse plant species. This conservation pattern suggests that AtBAM9 may function as an inducible regulator of starch degradation, modulated by circadian rhythms and responsive to environmental stimulates [9]. Both AtBAM7 and AtBAM8 are nucleus-localized transcription factors that contain a BRASSINAZOLE-RESISTANT1 (BZR1)-type DNA binding domain, influencing shoot growth and development via interactions with the brassinosteroid signaling pathway [5]. Thus, the *BAM* gene family members exhibit functional complexity.

To date, genome-wide analyses have identified a varying number of *BAM* genes across different plant species: six in grapevine (*Vitis vinifera*) [10], seven in Atemoya (*Annona atemoya*) [11], eight in trifoliate orange (*Poncirus trifoliata*) [12], nine in jujube (*Ziziphus jujuba*) [13], ten each in cassava (*Manihot esculenta*) [14] and rice [15], sixteen in banana (*Musa acuminata*) [16] and kiwifruit (*Actinidia arguta*) [17], seventeen in Chinese white pear (*Pyrus bretschneideri*) [18], twenty-one in apple (*Malus domestica*) [19] and white clover (*Trifolium repens*) [20], and twenty-seven in upland cotton (*Gossypium hirsutum*) [21]. Despite this, comprehensive information regarding the *BAM* gene family in pomegranate (*Punica granatum*) remains limited.

Pomegranate (*Punica granatum* L.), an ancient perennial species indigenous to Central Asia, is commercially cultivated across over 30 countries, including India, Iran, Spain, China, and the United States. With a history of cultivation exceeding 2000 years, China is recognized as one of the leading global producers of pomegranates [22]. Pomegranate trees thrive in warm climates and have a low tolerance for cold temperatures, leading to their predominant cultivation in tropical and subtropical regions [23]. Cold snaps during winter and late spring are prone to inducing freezing damage in pomegranate trees, which can significantly impair fruit yield and quality, consequently diminishing market availability. Consequently, temperature has emerged as a pivotal factor limiting the expansion of the pomegranate industry. Notably, the soft-seeded cultivar ‘Tunisia’ is the most widely planted in China, favored for its excellent taste, soft and edible kernel, and high nutritional value. However, it displays a heightened vulnerability to cold stress [24]. Cold temperatures below 3 °C can inflict damage on buds and new shoots, while temperatures descending below 10 °C hinder plant growth and development [24]. To date, several genes associated with cold stress tolerance in pomegranates have been characterized, including *PgICE1*, *PgCBF3*, and *PgCBF7* [25]. An RNA-seq analysis has uncovered that pathways related to starch and sucrose metabolism are significantly enriched in the response to cold and freezing stress of ‘Tunisia’ [24]. BAMs, known for their role in breaking down starch into soluble sugars, are integral to the accumulation of these sugars in response to cold stress. These soluble sugars are then translocated from the chloroplasts to the cytoplasm, where they engage in the energy metabolism pathway, bolstering the plant’s defense against cold-induced damage [26]. However, the precise members of the *BAM* family and their specific contributions to the pomegranate’s cold stress response have yet to be elucidated.

In this study, we analyzed eight *PgBAM* members at a genome-wide scale in pomegranate. In the analysis of phylogenetic relationships, gene structures, and conserved motifs among the eight *PgBAMs* and *BAM* members from seven other species, we observed a higher degree of conservation with the same group or subgroup. Furthermore, the syntenic analysis, combined with promoter prediction and expression pattern examination, revealed that *PgBAM4*, an ortholog of *AtBAM3*, is highly expressed in leaves and responds to cold stress, potentially regulated by PgCBF7. These findings advance our understanding of the potential roles of the *PgBAM* gene family members in pomegranate, particularly in their response to cold stress.

## 2. Results

### 2.1. Identification of the BAM Gene Family Members in Pomegranate

**Figure 1:**
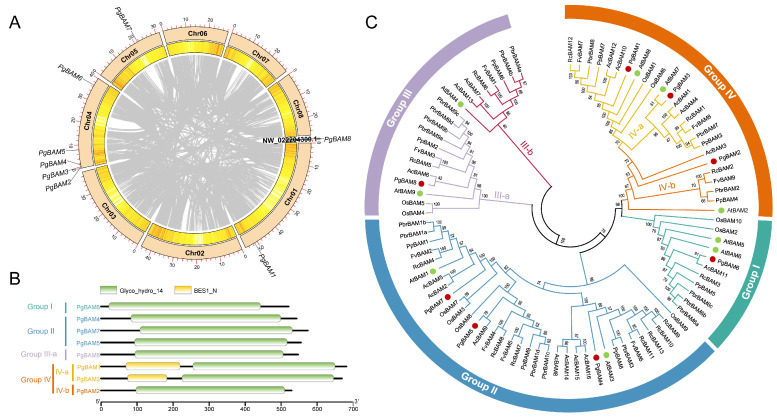
Chromosomal localization, conserved domain of *PgBAM* genes, and phylogenetic relationship among BAM members from pomegranate and seven other species. (**A**) Chromosomal localization and syntenic relationships of *PgBAM* genes in pomegranate. Gray lines mean homologous gene pairs. (**B**) Conserved domain identification of eight PgBAM members. (**C**) Phylogenetic analysis of BAMs from eight species. Red and green dots highlight pomegranate *PgBAM* and Arabidopsis *AtBAM* members. Phylogenetic tree was generated by NJ method and 1000 bootstrap replications using MEGA X. Arcs marked with cyan, blue, purple, and orange indicate different groups, respectively. Species names are abbreviated as follows: At, *Arabidopsis thaliana*; Os, *Oryza sativa*; Pg, *Punica granatum*; Ac, *Actinidia chinensis*; Pbr, *Pyrus bretschneideri*; Pp, *Prunus persica*; Rc, *Rosa chinensis*; and Fv, *Fragaria vesca*.

To identify members of the *BAM* gene family, BLASTP comparison and HMM searches were performed against the pomegranate database. After eliminating redundancy and confirming conserved domains, eight *PgBAM* members were identified. According to their chromosomal location order, these *PgBAM* members were named *PgBAM1*–*PgBAM8* (Figure 1A). Five of these genes were mapped to chromosome 04. No gene duplication events were detected within the pomegranate *BAM* gene family (Figure 1A). All members possessed a glycosyl hydrolase family 14 domain (397–426 aa), with PgBAM1 and PgBAM3 additionally containing an N-terminal BES1 domain (Figure 1B). A total of 91 BAM genes collected from pomegranate and seven other species were classified into four groups (Figure 1C; Table S1). Group III and IV, in particular, could be further divided into two subgroups each. Group II was the largest, comprising 35 members, followed by 18 in IV-a, 12 in III-a, 11 in group I, 8 in III-b, and 7 in IV-b. In pomegranate, three *PgBAM* members were categorized in group II, while group IV also contained three members. In contrast, group I and group III each contained a single *PgBAM* member (Figure 1C).

A physicochemical analysis and subcellular localization predictions indicated that 94.4% (17 members) of subgroup IV-a were likely to be localized in the nucleus (Table S3). These proteins exhibited a longer average length (673.7 aa) and a higher molecular weight (MW, 71.1 kDa) compared to the BAM proteins from other groups or subgroups. Moreover, approximately 68.6% of the members in group II were predicted to primarily function in the chloroplast. All the members of group I and IV and subgroup III-a were acidic. Likewise, in pomegranate, PgBAM1 and PgBAM3, which both contain the BES1 domain (Figure 1B), were also predicted to be nuclear-localized (Table 1). These proteins had a longer average length and higher MW than the other six PgBAM proteins. The remaining six PgBAM members showed less variation in protein length (528–573 aa) and MW (58.6–63.5 kDa) but exhibited a broader range of theoretical isoelectric points (pI, 5.33–8.84) and diverse subcellular localizations, suggesting their involvement in a variety of cellular functions.

### 2.2. Conserved Motif and Gene Structure Analysis of BAM Gene Family in Pomegranate, Arabidopsis, and Another Six Species

**Figure 2:**
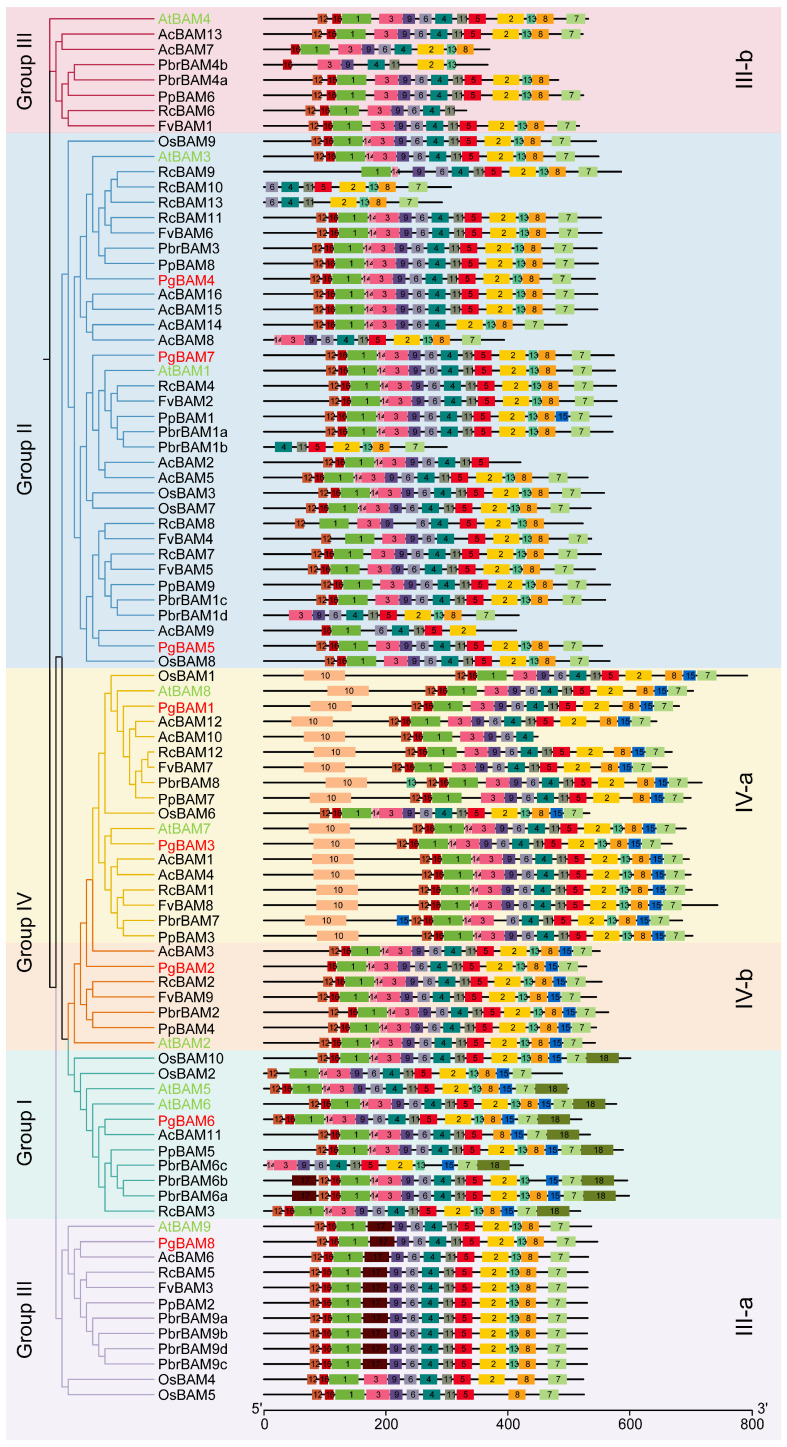
Composition and distribution of conserved motifs of BAM members. Eighteen motifs marked with different colors were identified using MEME web server. Members of pomegranate *PgBAM* and *Arabidopsis AtBAM* are highlighted in red and green, respectively.

A total of eighteen conserved motifs were identified across 91 BAM members using MEME webtools (Figure 2). The sequences and lengths of 18 motifs are listed in Table S4. The majority of motifs exhibited consistent composition and arrangement across different groups, which may reflect the conserved domains within the BAM family. Notably, motifs 12, 16, 1, 3, 9, 6, 4, 11, 5, 2, 13, 8, and 7 demonstrated a widespread presence and may be correlated with conserved functional domains (Figure 2). Furthermore, certain motifs were specifically observed in particular groups or subgroups. For example, motif 10 was predominantly found in IV-a, potentially linking to the distribution of the BZR1/BES1 domain. Motif 14 was frequently detected in group I and IV-b, as well as in 22 members of group II and 9 members of IV-a. Motif 15 was ubiquitously presented in groups I, IV-a, and IV-b, while motif 17 was exclusively identified in III-a, and motif 18 was specifically localized in group I. These motifs are potentially associated with group- or subgroup-specific functional divergence.

**Figure 3:**
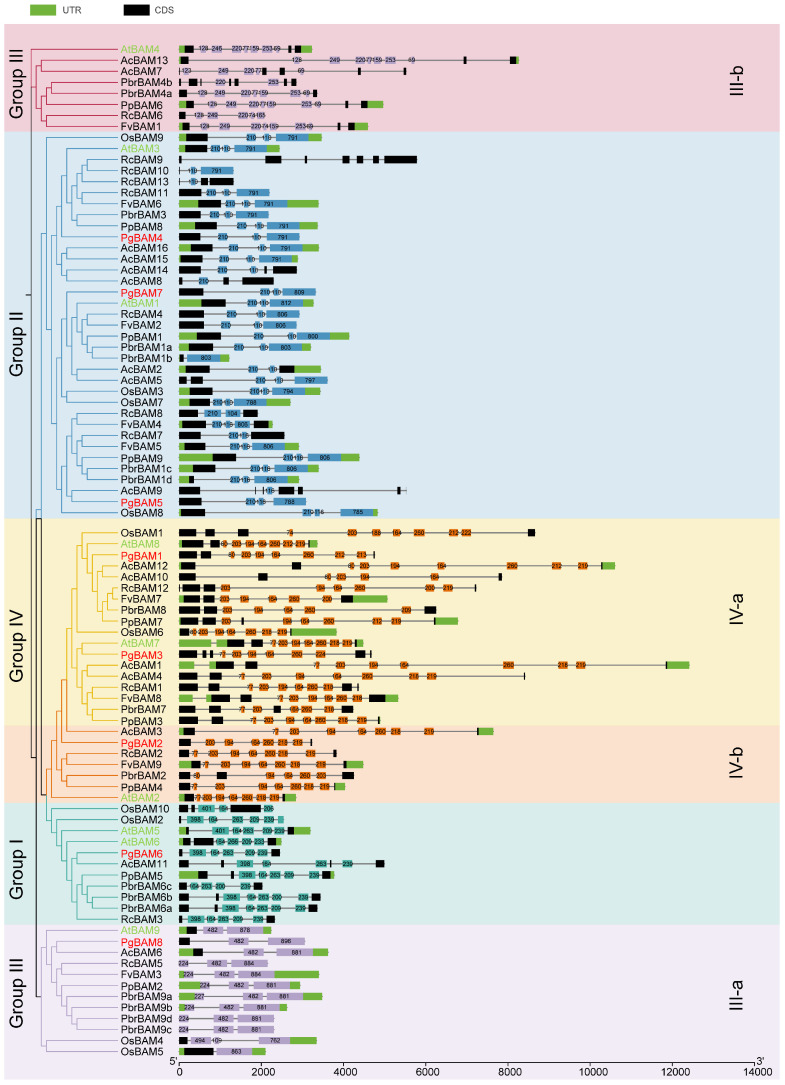
Exon organization of 91 *BAM* members from eight species. Exons and untranslated regions (UTRs) are marked with dark and green rectangles, respectively. Core exons shared within corresponding phylogenetic groups or subgroups are indicated by cyan, blue, purple, and orange rectangles. Numerical labels on exon rectangles correspond to exon lengths. Red and green highlight the members of pomegranate *PgBAM* and *Arabidopsis AtBAM*, respectively. All members were classified into four groups according to phylogenetic relationship.

In contrast to the conservation motifs observed among the four groups (Figure 2), significant variations in the number and composition of exons were noted among different groups or subgroups (Figure 3). The details of the exon lengths and arrangements for the 91 BAM members are presented in Table S5. For instance, in group II, 28 *BAM* members (80.0%) typically contained four exons. In subgroup III-a, 11 *BAM* genes (91.7%) possessed three exons, while subgroup III-b members (7, 87.5%) predominantly had at least nine exons. Group I included 11 genes with six to eight exons, and in group IV, 21 members (84.0%) had eight to ten exons. Furthermore, within the same group or subgroup, certain exons displayed consistent or similar lengths (Figure 3). In the majority of group I genes, five exons with lengths of 398 (or 401), 164, 263, 209, and 239 nucleotides (nt) were conserved and arranged in the same order. In group II, three exons with lengths of 210, 110 (or 116), and 791 (or 785, 788, 794, 800, 803, 806, 809, 812) nt were frequently observed. Subgroup III-a showed the conservation of exons with lengths of 224 (or 227), 482, and 881 (or 884, 878, 896, 863) nt, while subgroup III-b was characterized by exons of lengths 128, 249 (or 246), 220, 77 (or 74), 159 (or 165), 253, and 69 nt. In group IV, highly conserved exons with lengths of 77 (or 74, 80), 203, 194, 164, 260, 218 (or 212, 200, 209, 224), and 219 nt were arranged in a consistent order. Therefore, the gene structure exhibits distinct features across various groups and subgroups.

### 2.3. BAM Members’ Collinear Relationship and Protein Similarity Analysis

**Figure 4:**
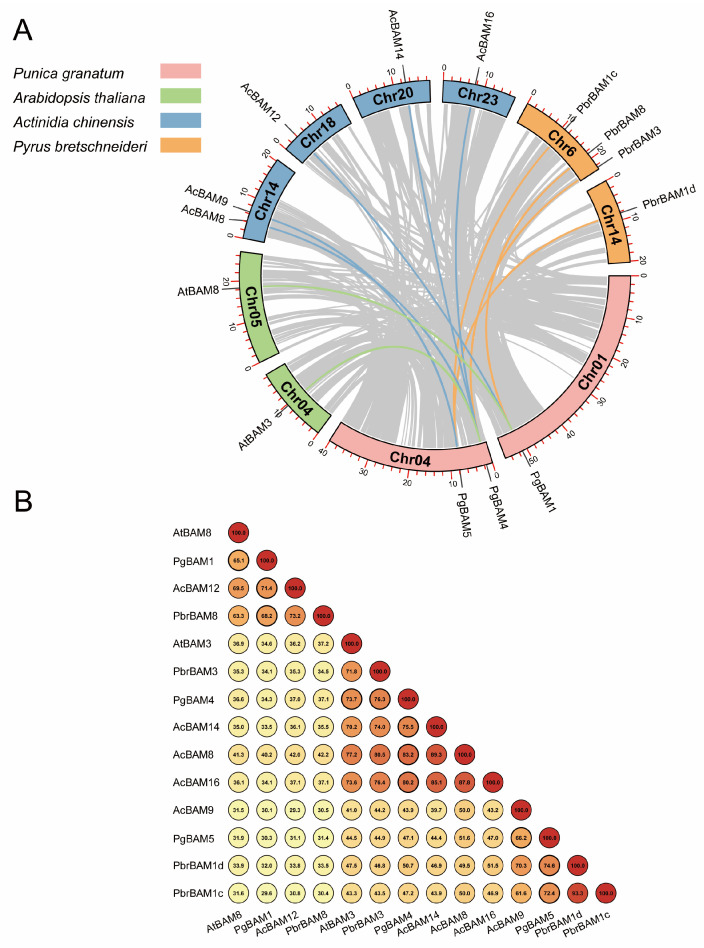
The syntenic relationship and sequence similarity of *BAM* members in pomegranate and three other species. (**A**) The syntenic analysis of *PgBAMs* with *Arabidopsis*, kiwifruit, and Chinese white pear. Gray lines represent homologous gene pairs, and colored curves illustrate the syntenic relationships between *BAM* members across species. (**B**) The protein sequence similarity among 14 BAM members. The larger number and the deeper red color in the circle indicate the higher similarity degree between the two sequences. The eleven bold circles highlight the sequence similarity of 11 homologous gene pairs.

To evaluate the homologous evolutionary relationships among *BAM* members of pomegranate, Arabidopsis, kiwifruit, and Chinese white pear, a synteny map was generated (Figure 4). In total, 11 orthologous gene pairs were identified between pomegranate and the three other examined species (Figure 4A). Specifically, *PgBAM4* had five orthologous gene pairs, while *PgBAM1* and *PgBAM5* each had three, which is consistent with their phylogenetic relationship (Figure 1). Moreover, these orthologous gene pairs displayed a high degree of sequence similarities (Figure 4B). For example, the orthologous genes were quite similar to PgBAM4, with protein similarities ranging from 74.0% (AtBAM3) to 83.2% (AcBAM8). These results imply that PgBAM4 may share similar gene functions with its orthologous genes.

### 2.4. Analysis of cis-Acting Elements in the Promoters of Eight PgBAM Genes

**Figure 5:**
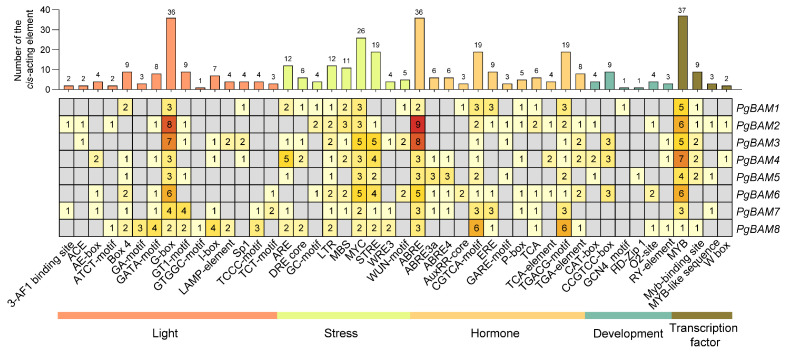
The analysis of the putative *cis*-acting elements in the promoter region of eight *PgBAM* genes. The 2000 nt was extracted for the *cis*-acting element analysis using the PlantCARE website.

*Cis*-acting elements, which are specific motifs located within the gene promoter region, play a crucial role in determining the initiation and level of gene expression. The promoters of eight *PgBAM* genes were extracted and analyzed to predict *cis*-acting elements using PlantCARE (Figure 5). A total of 394 elements were categorized into five main categories: light-responsive elements (98), stress-responsive elements (99), hormone-induced elements (124), development-related elements (22), and transcription factor binding elements (19). The hormone-responsive elements constituted the largest group, including 48 abscisic acid (ABA)-responsive elements (ABRE, ABRE3a, and ABRE4) discovered in the promoter regions of all *PgBAM* members. It was followed by 38 jasmonate-responsive elements (CGTCA-motif and TGACG-motif), 11 auxin-responsive elements (AuxRR-core and TGA-element), 10 salicylic acid (SA)-responsive elements (TCA-element), 9 ethylene-responsive elements (ERE), and 8 gibberellic acid (GA)-responsive elements (GARE-motif and P-box). The upstream regions of *PgBAM* also contained various stress-responsive elements, including drought-response elements (MBS and MYC), stress response element (STRE), low-temperature response elements (LTR), anaerobic induction (ARE and GC-motif), wounding responsiveness (WRE3 and WUN-motif), and dehydration-responsive elements (DRE). Multiple light-responsive elements were also predicted in the promoter region of *PgBAM*, indicating the essential role of light in regulating *PgBAM* transcription. In contrast, only 5.6% of the development-related elements were detected. Moreover, several transcription factor binding elements were identified, such as MYB, Myb-binding site, MYB-like sequence, and W box, which potentially participate in the regulation of *PgBAM* expression.

### 2.5. Expression Characteristic of PgBAM Family Genes in Different Plant Organs and Tissues

**Figure 6:**
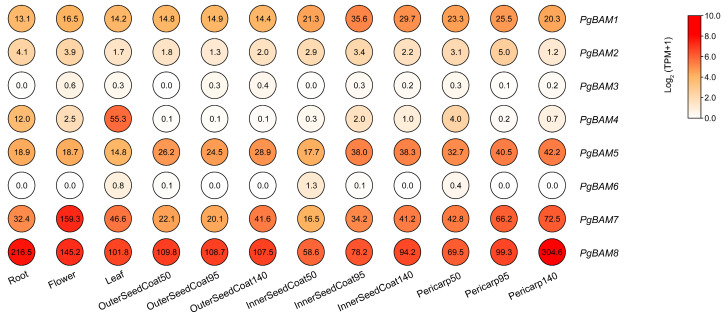
Expression characteristic of the *PgBAM* genes in different organs and tissues of pomegranate. The expression levels were detected in the root, flower, leaf, three development stages of the pericarp, and the inner and outer seed coats (50, 95, and 140 DAF). The expression levels are depicted in various degrees of red based on log_2_ (TPM + 1). The TPM values are correspondingly marked on each circle.

Based on the published RNA-seq data [27], we investigate the gene expression pattern of *PgBAM* members in different organs and tissues (Figure 6). *PgBAM1* exhibited relatively consistent expression across the root, flower, and leaf, and during the development of the outer seed coat and pericarp. In contrast, *PgBAM4* showed a higher level of expression specifically in the leaf when compared to other organs or tissues. *PgBAM5* and *PgBAM7* showed similar expression patterns during the development of the inner seed coat, while *PgBAM7* may also potentially play a role in the development of the outer seed coat and pericarp at 140 days. Notably, *PgBAM7* was predominantly expressed in the flower. On the other hand, *PgBAM8* showed high expression levels across all samples. For instance, its expression in the root and leaf was 11.3- and 3.1-fold higher than the average expression of *PgBAM1*/*4*/*5*/, respectively. During fruit ripening, the expressions of *PgBAM8* steadily increased within development of the inner seed coat and pericarp, particularly in the pericarp, where it was upregulated by 1.43- and 4.38-fold at 95 and 140 days, respectively, compared to its expression level at 50 days. However, *PgBAM2*/*3*/*6* in these detected samples, as well as *PgBAM4* during fruit ripening, were either not expressed or expressed at trace levels.

### 2.6. Transcriptome Analyses of ‘Tunisia’ Pomegranate for Response to Cold Stress

**Figure 7:**
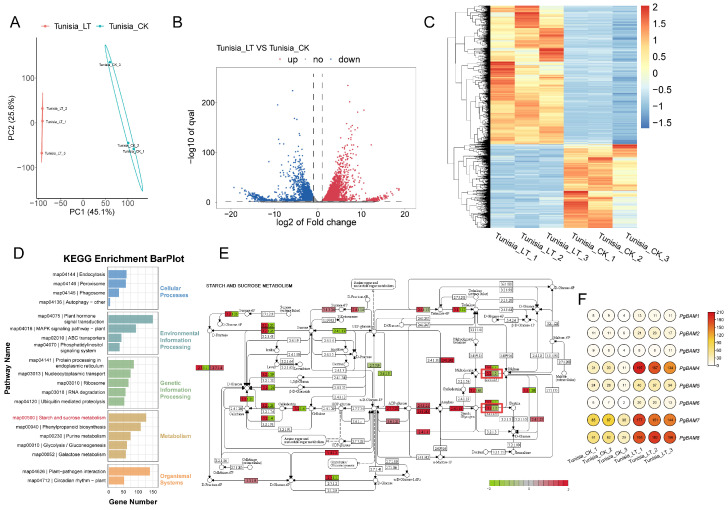
The transcriptome analysis of the cold stress response in the ‘Tunisia’ pomegranate. (**A**) The PCA analysis based on the TPM values of all genes in each sample. (**B**) The distribution of DEGs from the RNA-seq analysis. The *X*-axis represents the fold change in gene expression between Tunisia_LT and Tunisia_CK. The *Y*-axis represents the negative log_10_ (*p* value) for differences between the samples. (**C**) The cluster analysis of DEGs under different treatments. The intensity of the red coloration indicates the degree of gene upregulation; similarly, the intensity of the blue coloration signifies the degree of gene downregulation. Horizontal is the expression level of DEGs, and each column is the sample. (**D**) KEGG pathways of DEGs in Tunisia_LT versus Tunisia_CK. (**E**) The KEEG pathway of starch and sucrose metabolism (map00500). (**F**) The expression of eight *PgBAM* genes. The expression levels are depicted in various degrees of red based on log_2_ (TPM + 1). The TPM values are correspondingly marked on each circle. CK and LT denote the seedling leaves subjected to low-temperature treatment for 0 and 12 h, respectively.

The pomegranate variety ‘Tunisia’, characterized by its soft seeds, is known to be sensitive to cold stress. To investigate the genes’ expression in response to cold stress, an RNA sequencing analysis was performed on seedling leaves treated with a low temperature (LT) (Figure 7). A principal component analysis (PCA) revealed that the six sample libraries segregated into two distinct groups, corresponding to their respective treatments (Figure 7A). Subsequently, a significant number of differentially expressed genes (DEGs) were identified, with 4564 genes upregulated and 2754 genes downregulated (Figure 7B). Consistent with the PCA findings, DEGs displayed comparable expression patterns within their respective treatment groups (Figure 7C). The details of DEGs have been provided in Table S6. Furthermore, based on the KEGG enrichment analysis, the DEGs were classified into five main categories and 20 pathways (Figure 7D). Within these pathways, a total of 127 DEGs were involved in the starch and sucrose metabolism pathway (00500), including five *PgBAM* members (*PgBAM2*/*4*/*6*/*7*/*8*), suggesting their potential roles in starch and sucrose metabolism (Figure 7E). Notably, the expression of *PgBAM4* increased by 6.65-fold in response to LT stress (Figure 7F).

### 2.7. PgBAM4 May Be a Key Candidate for Cold Stress Response

**Figure 8:**
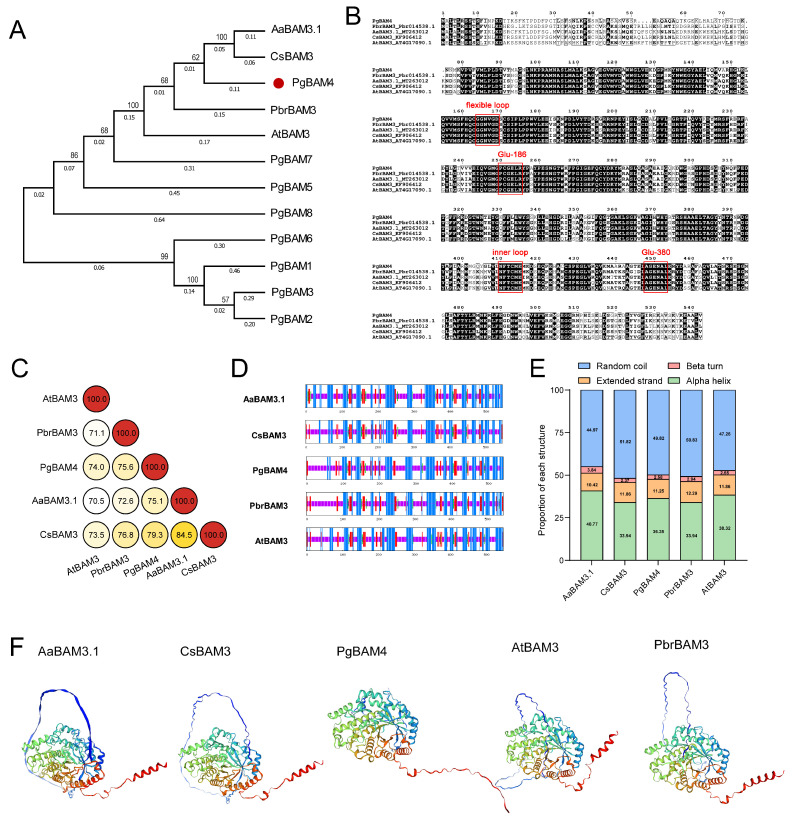
The evolutionary and protein sequence analysis between PgBAM4 and the cold-tolerance-associated BAM genes based on the phylogenetic relationship, protein sequence alignment, and similarity, as well as secondary and tertiary structures. (**A**) The phylogenetic relationship analysis among eight PgBAMs, AtBAM3, PbrBAM3, AaBAM3.1, and CsBAM3. (**B**) Protein sequence alignment of PgBAM4, AtBAM3, PbrBAM3, AaBAM3.1, and CsBAM3. Multiple sequence alignment was performed using the ClustalOmega web service. Red boxes represent the amino acid motifs that are crucial for BAM catalytic activity, including the flexible loop, inner loop, Glu-186, and Glu-380. (**C**) Protein sequence similarity among PgBAM4, AtBAM3, PbrBAM3, AaBAM3.1, and CsBAM3. (**D**,**E**) The protein secondary structure distribution and proportion prediction of AaBAM3.1, CsBAM3, PgBAM4, PbrBAM3, and AtBAM3. (**F**) The tertiary structure prediction of AaBAM3.1, CsBAM3, PgBAM4, AtBAM3, and PbrBAM3 using the Swiss model.

Several genes, classified along with AtBAM3, have been demonstrated or predicted to regulate cold tolerance [26,28,29,30]. A phylogenetic relationship analysis indicated that PgBAM4 shares a close evolutionary relationship with the cold-responsive *BAM* genes (AaBAM3.1, CsBAM3, PbrBAM3, and AtBAM3) (Figure 8A). Both PgBAM4 and these cold-responsive BAM members exhibited highly conserved amino acid motifs that are crucial for the catalytic activity of β-amylase. These essential motifs include the catalytic residues Glu-186 and Glu-380, as well as the flexible loop and inner loops (Figure 8B) [4]. Additionally, PgBAM4 also shared high sequence similarities (76.0% identity on average) with the cold-responsive BAM genes (Figure 8C). The protein secondary structures, the proportional distribution of each structure, and the tertiary structures of PgBAM4 and these cold-responsive BAM genes were similar, implying that PgBAM4 may contribute to cold tolerance in pomegranate (Figure 8D–F).

### 2.8. PgCBF7 May Play a Role in Regulating PgBAM4 Expression for Cold Response

**Figure 9:**
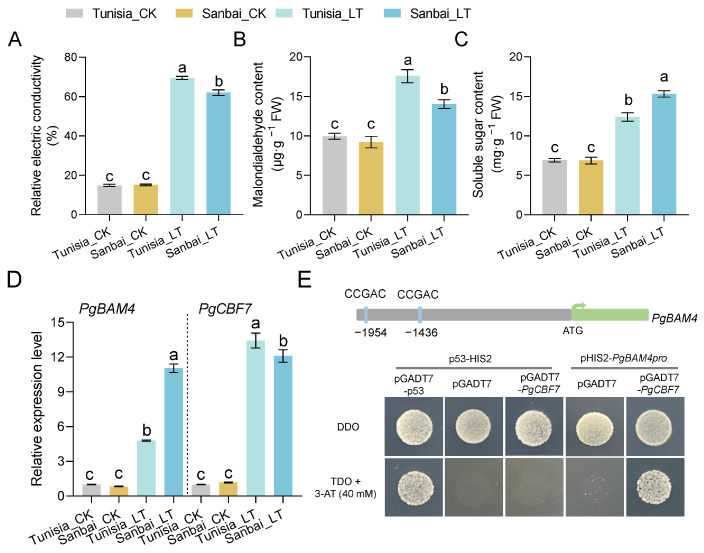
PgCBF7 may be involved in *PgBAM4* responding to cold stress among different pomegranate cultivars. The analysis of the effect of cold stress on relative electric conductivity (**A**), MDA (**B**), and soluble sugar content (**C**), and the expression of *PgBAM4* and *PgCBF*7 between ‘Tunisia’ and ‘Sanbai’ (**D**). (**E**) The bind activity of PgCBF7 on the promoter region of *PgBAM4* was measured using Y1H. Data are the means of three replicates (±SE). Different letters indicate statistically significant differences between the samples (one-way ANOVA, followed by a post hoc Tukey test, *p* < 0.05). CK and LT denote the seedling leaves subjected to low-temperature treatment for 0 and 12 h, respectively.

To assess the role of *PgBAM4* in the cold stress response, we detected its expression in two pomegranate cultivars, ‘Tunisia’ and ‘Sanbai’. The results of REC, MDA, and soluble sugar content reflected that ‘Sanbai’ exhibited greater cold stress tolerance compared to ‘Tunisia’ (Figure 9A–C). Specifically, ‘Sanbai’ showed significantly lower REC and MDA content than ‘Tunisia’ when exposed to LT (Figure 9A,B). Conversely, ‘Sanbai’ had a 1.24-fold higher soluble sugar content than ‘Tunisia’ under LT (Figure 9C). Correspondingly, the expression of *PgBAM4* in ‘Sanbai’ significantly increased by 11.0-fold under LT compared to room temperature (CK), surpassing the expression levels in ‘Tunisia’ under LT by 2.31-fold (Figure 9D). These results suggest that *PgBAM4* may be a key gene for cold stress tolerance, with its elevated expression potentially contributing to increased soluble sugar content and enhancing the cold stress tolerance in ‘Sanbai’.

Additionally, the expression of *PgCBF7* (LOC116202526), a known transcription factor crucial for pomegranate’s cold response [25,31], was also detected. Despite differences in cold stress tolerance, *PgCBF7* was significantly upregulated under LT in both ‘Tunisia’ and ‘Sanbai’ cultivars (Figure 9D), confirming its pivotal role in regulating cold responses. Two putative DRE-core elements (GCCGAC) within the promoter regions of *PgBAM4* at −1436 and −1954 bp were identified, suggesting the potential regulatory role of CBFs/DREB1s’ transcription factors (Figure 9E). The yeast one-hybrid assay (Y1H) results showed that the transformants carrying the pHIS2-*PgBAM4pro* plasmid were not able to grow on SD/-Trp/-His media supplemented with 40 mM of 3-AT (Figure S1). The screening was conducted on a TDO medium supplemented with 40 mM of 3-AT (Figure 9E). The yeast cells transformed with pHIS2-*PgBAM4pro* + pGADT7-*PgCBF7*, similar to the positive control cells harboring p53-HIS2 + pGADT7-53, exhibited growth on the TDO medium containing 40 mM 3-AT. In contrast, the negative control yeast cells (p53-HIS2 + pGADT7, p53-HIS2 + pGADT7-*PgCBF7*, and pHIS2.1-*PgBAM4pro* + pGADT7) only showed growth on the DDO medium. These observations showed that PgCBF7 has the ability to bind to the *PgBAM4* promoter. Therefore, the results indicate that PgCBF7, acting as a transcription factor, may be involved in regulating the expression of *PgBAM4* during the cold response.

## 3. Discussion

In plants, β-amylase primarily catalyzes the hydrolysis of starch. The availability of extensive genomic data from various species has greatly facilitated the identification of *BAMs’* gene families. Multiple copies of *BAM*-like sequences were identified in different land plant species [4]. All these members were clustered into four groups, according to sequence similarities and gene structural features [2,13,16]. However, it is hypothesized that the earliest land plants possessed only two subfamilies of *BAM* genes, which diverged before the emergence of land plants [5]. The rest of the *BAM* genes likely evolved from these ancestral genes through individual gene duplication or WGD events [5]. For instance, in *Arabidopsis*, the members *AtBAM5*/*6* within subfamily I and *AtBAM2*/*7* within subfamily IV are the result of a recent duplication event [4]. However, the protein sequence similarity among all AtBAMs was less than a 45% identity [5]. In this study, we identified a total of eight PgBAM members in the pomegranate genome (Figure 1A). Notably, no gene duplication events were identified (Figure 1A). These identified PgBAM members exhibited a relatively low protein sequence similarity (41.4%). This study suggests that none of the *PgBAM* or *AtBAM* members have resulted from very recent gene duplications [2].

The phylogenetic analysis revealed that the 91 *BAM* candidates are categorized into four distinct groups, consistent with the classification of *BAM* orthologs in *Arabidopsis* (Figure 1C) [2]. All members exhibited a consistent arrangement of at least 13 conserved motifs (Figure 2). In contrast, notable variations in gene structure, protein length, molecular weight, theoretical isoelectric point, and predicted cellular locations were observed among different phylogenetic groups or subgroups in *Arabidopsis* [2], rice, pomegranate (Figure 1C; Table 1; Figure 3), kiwifruit, Chinese white pear [18], peach, rose, woodland strawberry, jujube [13], and banana [16]. These results confirm that the complexity of the β-amylase gene structure and function is a shared characteristic among higher plants, and not unique to *Arabidopsis* [2]. Within the same phylogenetic group or subgroup, members shared a similar gene structure and arrangement of conserved motifs (Figure 2 and Figure 3) [2]. These findings indicate that the ancient evolution of the different *BAM* groups occurred earlier than the separation of these species.

*BAM* modulates starch degradation and is involved in regulating plant growth, development, and abiotic stress response [2,3]. For fruit crops, starch degradation is an important factor for fruit quality during ripening. Previous studies have indicated that members of the BAM family, particularly AtBAM9, are likely to be involved in starch metabolism during fruit development. For example, the expression of *AdBAM9* (FG460922 in NCBI, *AcBAM6* in *Actinidia chinensis* Hong Yang v3) peaked early in the stages of kiwifruit development, exhibiting a pattern that corresponds to the changes in glucose content [32]. Similarly, *ZjBAM9*, which clusters with *AtBAM9*, was significantly upregulated in jujube fruit [13]. Banana *MaBAM9b* (Ma05_t07800.1), grouped with *AtBAM9* [16], displayed high expression levels that were positively correlated with starch degradation and ripening in banana fruit [33]. In our study, *PgBAM8*, which is classified in the same subgroup, subgroup III-a, as *AtBAM9* (Figure 1C), showed a conserved motif arrangement and a similar exon distribution (Figure 2 and Figure 3). Notably, the expression of *PgBAM8* was upregulated in pericarp during pomegranate fruit development (Figure 6). These findings suggest that *PgBAM8* may play a conserved role among the members of the same group or subgroup during fruit development, which needs a further investigation.

The starch and sucrose metabolism pathway plays a pivotal role in plant abiotic stress responses, including salt [34,35], drought [36], high temperature [37], and cold [38,39,40]. This pathway contributes to the accumulation of soluble sugars, thereby increasing cellular permeation concentration and water potential, and ultimately enhancing stress tolerance. In our study, the starch and sucrose metabolism pathway was prominently identified in the transcriptome analysis (Figure 7D), and the soluble sugar content was significantly increased under cold stress (Figure 9C), supporting the findings of previous research [24]. As a member of the starch and sucrose metabolism pathway, β-amylase also acts as a regulator in the stress response. Specifically, BAMs within group II, which are orthologous to *AtBAM*, have played universal roles when plants are subjected to LT stress. In *Arabidopsis*, *AtBAM3* (AtBMY8) was specifically induced by a cold shock, and its knockdown results in reduced levels of maltose, glucose, fructose, and sucrose, increasing sensitivity to freezing stress [7]. Similarly, *PbrBAM3* positively contributed to cold stress tolerance in pear by elevating soluble sugar levels and activating the system for scavenging ROS [30]. In trifoliate orange, *PtrBAM1*, closely related to *AtBAM3*, was verified regarding its location in the chloroplast and its overexpression in tobacco enhances tolerance to chilling and freezing temperatures through increased BAM activity and soluble sugar accumulation [12]. *AaBAM3.1* in kiwifruit, classified with *AtBAM3*, has been shown to enhance freezing tolerance [26]. In tea plants, *CsBAM3* was putative as a key gene encoding β-amylase, which was involved in starch metabolism and the response to cold stress [29]. In grape, the expression of *VvBAM3* increased gradually under chilling conditions, reflecting its role in starch breakdown at a low temperature [41]. Recently, *TrBAM04*/*09*/*10*, as the homologous genes to *AtBAM3*, were notably upregulated under cold stress in white clover [20]. In our study, a close relationship was identified between PgBAM4 and AtBAM3, with these orthologous genes exhibiting high protein sequence similarity (Figure 4). Notably, they and other cold-responsive BAM genes (AaBAM3.1, CsBAM3, and PbrBAM3) shared conserved amino acid motifs that are known to be crucial for β-amylase activity. Moreover, PgBAM4, AtBAM3, and these cold-responsive BAM genes display similar secondary and tertiary structures (Figure 8). The elevated expression level of *PgBAM4* was observed under LT stress both in RNA-seq and RT-qPCR analyses (Figure 7F and Figure 9D). These results suggest that *PgBAM4* may share a conserved function with *AtBAM3* and other cold-responsive *BAM* genes in accelerating starch degradation and increasing soluble sugar content. Furthermore, our results demonstrated a positive correlation between high *PgBAM4* expression, soluble sugar content, and cold tolerance in the ‘Sanbai’ variety (Figure 9A–C). Additionally, DRE-core elements, which bind to CCGAC core sequences via CBFs in response to LT stress [42], may play a role in the regulation of *PgBAM4*. In kiwifruit, AaCBF4 has been shown to directly bind to the DRE *cis*-element in the promoter of *AaBAM3.1*, activating it under cold stress and enhancing freezing tolerance [26]. Likewise, in pomegranate, PgCBF7, a crucial transcription factor responding to cold stress stimulation (Figure 9D) [25,31], is likely to regulate the expression of *PgBAM4* by binding to the DRE-core element in its promoter region (Figure 9E). Our findings suggest that *PgCBF7*-*PgBAM4* may play a role in positively regulating the low-temperature tolerance of pomegranate.

## 4. Materials and Methods

### 4.1. Plant Material and Treatment

Two different cold-stress-tolerant pomegranate cultivars, ‘Tunisia’ and ‘Sanbai’, were planted in the greenhouse of the horticultural experimental station of Huaibei Normal University, Huaibei, China. Cuttings, without the application of any additional rooting hormones, were cultured in a growth chamber at 22 °C and 65% humidity under a 14 h/10 h light/dark photoperiod. Healthy and uniform two-month-old pomegranate cuttings were subjected to cold stress in a growth chamber set at −4 °C. Leaf samples were collected at 0 and 12 h after the treatment. At each time point, three replicates of samples were collected, immediately frozen in liquid nitrogen, and subsequently stored in a −80 °C refrigerator.

### 4.2. BAM Gene Family Identification in Pomegranate

The genomic dataset for the pomegranate cultivar ‘Tunisia’ (*Punica granatum* L.), version ASM765513v2, was downloaded from the NCBI (https://www.ncbi.nlm.nih.gov/datasets/genome/GCF_007655135.1/, accessed on 20 December 2023) [43]. The Chinese white pear (*Pyrus bretschneideri*) was obtained from Pear Multiomics Database (http://pearomics.njau.edu.cn/, accessed on 20 December 2023). The Hong Yang kiwifruit (*Actinidia chinensis*) genome data were collected from KGD (http://kiwifruitgenome.org/, accessed on 20 December 2023). The *BAM* members of AtBAMs and OsBAMs were downloaded from TAIR (https://www.arabidopsis.org, accessed on 20 December 2023) and the Rice genome annotation project website (http://rice.uga.edu, accessed on 20 December 2023), respectively. Nineteen BAM members from both AtBAMs and OsBAMs were used as queries to perform a local BLASTP search against the pomegranate database for PgBAM candidate identification (E-value ≤ 1 × 10^−5^). The hidden Markov model (HMM) profiles of Glyco_hydro_14 (PF01373) were obtained from the InterPro database (https://www.ebi.ac.uk/interpro/entry/pfam/PF01373/curation/, accessed on 20 December 2023) and used as the query to search the PgBAM candidates using HMMER 3.1 (E-value ≤ 1 × 10^−5^). The candidates were verified using SMART (http://smart.embl-heidelberg.de/, accessed on 20 December 2023) to confirm the presence of the conserved domains. All protein sequences of 91 BAM members are listed in Table S1.

The length of amino acids, molecular weight, and isoelectric point were calculated using the ExPasy ProtParam tool (http://web.expasy.org/protparam/, accessed on 20 December 2023). The BUSCA (http://busca.biocomp.unibo.it/, accessed on 20 December 2023) was employed for the prediction of subcellular localization.

The chromosomal positions and collinear relationships of eight *PgBAM* were analyzed and visualized using the TBtools’ plugin (One step MCScanX—Super Fast) and the “Advanced Circos” function in TBtools-II [44].

### 4.3. Phylogenetic Analysis and Classification of BAM Gene Family

A total of 91 BAM members, collected from *Arabidopsis*, rice, pomegranate, kiwifruit, Chinese white pear, peach, rose, and woodland strawberry, were utilized to generate a phylogenetic tree using MEGA X.1.6 software with the neighbor-joining method and 1000 bootstrap replicates. The members were classified into four groups according to their evolutionary relationships with *BAM* members of *Arabidopsis* and rice.

### 4.4. Conserved Motif and Gene Structure Analysis of BAM Gene Family

The online MEME program (https://meme-suite.org/meme/tools/meme, accessed on 20 December 2023) was used to analyze the composition of conserved motifs among 91 BAM members. The parameters were set as follows: motif site distribution to allow any number of repetitions (anr), a maximum of 18 motifs, and motif lengths ranging from 6 to 200. The gene structure data of 91 BAM members were acquired from their genome annotation files, respectively. The results were visualized using TBtools-II.

### 4.5. Collinear Relationships and Sequence Similarity Analysis

The collinear relationships of *BAM* members among *Arabidopsis*, pomegranate, kiwifruit, and Chinese white pear were analyzed using the TBtools-II plugin (One step MCScanX—Super Fast) with default parameters. The results were visualized as a circos plot according to the “Advanced Circos” function within TBtools-II. The similarity among the protein sequences was calculated using Clustal Omega (https://www.ebi.ac.uk/Tools/msa/clustalo/, accessed on 20 December 2023).

### 4.6. Cis-Acting Element Analysis of PgBAM Genes

The putative promoter sequences of *PgBAMs* (2000 nucleotides (nt) upstream of the start codon) were obtained from genomic sequences. The *cis*-acting elements were predicted using the PlantCARE website (https://bioinformatics.psb.ugent.be/webtools/plantcare/html/, accessed on 20 December 2023) with default parameters. The results were visualized as a heatmap using TBtools-II.

### 4.7. Expression Characteristics of PgBAM Genes

The transcriptome dataset from 12 samples, which included the root, leaf, flower, pericarp, and inner and outer seed coats at 50, 95, and 140 days after flowering (DAF), was utilized to analyze the expression patterns of eight *PgBAM* members. The raw RNA-seq data were downloaded from NCBI (accession number SRP100581, accessed on 20 December 2023) [27]. The data analysis and visualization were performed as previously described [45].

### 4.8. Transcriptome Sequence and Data Analysis

The samples of cold-sensitive cultivar ‘Tunisia’ treated with cold stress, 0 and 12 h, were used for transcriptome sequencing. Total RNA was extracted from each sample using a TRIzol reagent (ThermoFisher, 15596018, Waltham, MA, USA), following the manufacturer’s protocol. The RNA concentration and integrity were quantified and assessed using the NanoDrop ND-1000 (NanoDrop, Wilmington, DE, USA) and the Bioanalyzer 2100 (Agilent, Santa Clara, CA, USA), respectively. mRNA was particularly purified by oligo (dT) magnetic beads, subsequently used for cDNA library construction. A total of six RNA libraries were sequenced for 2 × 150 bp paired-end reads on the illumina NovaseqTM 6000 platform by LC Bio Technology CO., Ltd. (Hangzhou, China). Control samples, not subjected to cold stress (0 h), were named Tunisia_CK, with each of the three replicates designated as Tunisia_CK_1, Tunisia_CK_2, and Tunisia_CK_3. Samples exposed to low-temperature treatment for 12 h were named Tunisia_LT, with the three replicates correspondingly labeled as Tunisia_LT_1, Tunisia_LT_2, and Tunisia_LT_3.

After filtering the raw reads, clean reads were aligned to the reference genome of the pomegranate (ASM765513v2) using HISAT2 with default parameter settings [46]. StringTie was then utilized for transcript assembly of the mapped reads for each sample and to estimate the transcript expression levels by calculating TPM (transcripts per kilobase of exon model per million mapped reads) values [47]. Subsequent bioinformatic analyses and graph plotting were conducted using OmicStudio tools [48], which included a principal component analysis (PCA), differential expression analysis and volcano as well as heatmap plot, and KEGG enrichment analysis and pathway plot. To facilitate heatmap visualization, TPM values were transformed into log2 (TPM + 1) to plot the heatmap using TBtools-II. Genes were considered differentially expressed if they met the criteria of a false discovery rate (FDR) less than 0.05 and an absolute fold change ≥ 1. These differentially expressed genes were further subjected to an enrichment analysis of KEGG pathways.

### 4.9. Protein Structure Analysis

The secondary protein structures of PgBAM4, AtBAM3, PbrBAM3, and AcBAM3 were predicted using NPS@: SOPMA (https://npsa-prabi.ibcp.fr/cgi-bin/npsa_automat.pl?page=npsa%20_sopma.html, accessed on 20 December 2023). Additionally, the tertiary structural models for these proteins were predicted and visualized using ExPaSy Swiss-Model online software (https://swissmodel.expasy.org/, accessed on 20 December 2023).

### 4.10. Assay of Relative Electric Conductivity (REC), and the Content of Malondialdehyde (MDA) and Soluble Sugar

The REC values of ‘Tunisia’ and ‘Sanbai’ leaves, following 0 and 12 h of cold stress treatment, were determined according to the method described by Zhou and Leul [49]. Fresh leaf samples (1.0 g) were cut into 1 cm^2^ slices and placed in a test tube containing 10 mL of deionized water. The test tubes were vacuum-infiltrated for 10 min. Subsequently, the leaf samples were immersed and vibrated in a water bath at 25 °C for 20 min, and the initial conductivity of the solution (C1) was measured using a conductivity meter. After boiling the samples for 10 min and allowing them to cool to room temperature, the final conductivity (C2) was measured. REC (%) was calculated as C1/C2 × 100%.

The content of MDA in leaf samples was determined using the 2-thiobarbituric acid (TBA) assay. A total of 1 g of leaf samples was homogenized in 5 mL of a 10% (*w*/*v*) trichloroacetic acid (TCA) solution. After centrifugation at 10,000 rpm for 10 min, 2 mL of the supernatant was transferred and mixed with 2 mL of a 0.6% (*w*/*v*) TBA solution. The mixture was heated in boiling water for 15 min, and then quickly cooled to room temperature. The mixture was centrifuged again at 10,000 rpm for 10 min. The absorbance of the supernatant was measured at 532, 600, and 450 nm with a UV–vis spectrophotometer.

The soluble sugar content was quantified using the anthrone colorimetric methods [50]. Leaf tissue (0.1 g) was ground with 3 mL of 80% ethyl alcohol. After centrifugation at 5000 rpm for 10 min, 1 mL of the supernatant was mixed with 5 mL of a 0.1% (*w*/*v*) anthrone solution containing 98% concentrated sulfuric acid. The mixture was then incubated at 100 °C for 10 min. The solutions were rapidly cooled in cold water to room temperature. The absorbance of the reaction solution was measured at 620 nm.

### 4.11. RNA Extraction and Real-Time Quantitative PCR (RT-qPCR) Analysis

Total RNA was isolated from three biological replicates of each plant sample using the plant RNA extraction kit (Biofit, Chengdu, China) following the manufacturer’s protocol. Genomic DNA was eliminated, and cDNA was synthesized according to the instructions of TransScript (One-Step gDNA Removal and cDNA Synthesis Supermix kit, Transgen Biotech, Beijing, China). RT-qPCR was conducted using the ChamQ Universal SYBR qPCR Master Mix (Vazyme, Nanjing, China) kit to prepare the reaction mixture, which was analyzed with the ABI-QuantStudio6 Flex real-time fluorescence quantitative PCR instrument. The *PgActin* gene served as an internal reference for normalizing variations in gene expression. The relative expression levels of the target genes were quantified using the ΔΔCt method as described by Livak and Schmittgen [51]. Each treatment was subjected to three biological replicates. The primers used for RT-qPCR are listed in Table S2.

### 4.12. Yeast One-Hybrid (Y1H) Assay

A yeast one-hybrid (Y1H) assay was performed using a Y187-pHis2 Yeast One-Hybrid interaction proving kit (Coolaber, Beijing, China) following the manufacturer’s protocol. The promoter sequence (2000 bp) of *PgBAM4* was cloned from genomic DNA and ligated into the pHIS2 vector to construct a recombinant vector (pHIS2-*PgBAM4pro*). The open reading frame (ORF) of *PgCBF7* (LOC116202526) was inserted into the pGADT7 vector to create pGADT7-*PgCBF7*. The primers used in the Y1H assay are listed in Table S2. To identify the optimal concentration of 3-amino-1,2,4-triazole (3-AT) for suppressing background histidine leakiness of the pHIS2-*PgBAM4pro* vector, the recombinant vector was transformed into the Y187 yeast strain and cultured on an SD/-Trp/-His medium containing a range of 3-AT concentrations. The vectors p53-HIS2 and pGADT7-p53 served as positive controls, while negative controls were prepared by transforming with p53-HIS2 + pGADT7, p53-HIS2 + pGADT7-*PgCBF7*, or pHIS2-*PgBAM4pro* + pGADT7, respectively. Transformants were selected on a double dropout (DDO, SD/-Trp-Leu) medium and then plated onto a triple dropout (TDO, SD/-Trp-Leu-His) medium containing 40 mM 3-AT. The yeast cells were incubated at 28 to 30 °C for 48 to 72 h to assess growth.

## 5. Conclusions

Our study is the first comprehensive investigation of the pomegranate *BAM* gene family, with particular focus on the role of *PgBAM4* under cold stress conditions. We identified a total of eight *BAM* members in the pomegranate genome, which were classified into four groups. The PgBAM members showed high evolutionary conservation with BAM members of other species within the same group or subgroup. *PgBAM4* may share a common molecular function with the cold-responsive *BAM* genes in the starch and sucrose metabolism pathway, contributing to cold stress tolerance. Furthermore, this function may be potentially regulated by PgCBF7, although further verification is needed.

## Figures and Tables

**Table 1:** Molecular characteristics and prediction of subcellular localization of PgBAM members.

Gene Name	Gene ID	Group or Subgroup	Subcellular Localizations	Number of Amino Acids (aa)	Molecular Weight (kDa)	Theoretical Isoelectric Points
PgBAM1	LOC116191971	IV-a	nucleus	680	76.5	5.83
PgBAM2	LOC116204287	IV-b	chloroplast thylakoid membrane	528	59.9	5.33
PgBAM3	LOC116204286	IV-a	nucleus	668	75.1	5.35
PgBAM4	LOC116203731	II	chloroplast thylakoid membrane	542	60.3	8.75
PgBAM5	LOC116204822	II	mitochondrion	554	62.3	8.84
PgBAM6	LOC116202491	I	cytoplasm	520	58.6	6.13
PgBAM7	LOC116208025	II	chloroplast thylakoid membrane	573	63.5	5.97
PgBAM8	LOC116190185	III-a	mitochondrion	546	60.1	5.9

## Data Availability

The data presented in this study are available on request from the corresponding author.

## Supplementary Materials

The following supporting information can be downloaded at: https://www.mdpi.com/article/10.3390/plants13101321/s1, Figure S1: Screening of the optimal concentration of 3-AT used for the yeast one-hybrid assay; Table S1: BAM members’ protein sequence used in this study; Table S2: The primers used in this study; Table S3: Molecular characteristics and prediction of subcellular localization of the BAM proteins; Table S4: Sequence and length of eighteen motifs of 91 BAM members; Table S5: Exon length and arrangement of 91 members; Table S6: The detail of the differentially expressed genes.
