# Supplementary material for: A Genome-Wide Analysis of the BAM Gene Family and Identification of the Cold-Responsive Genes in Pomegranate (Punica granatum L.)
Source: Plants (Basel). 2024 May 10;13(10):1321. doi: 10.3390/plants13101321 (PMC11125002; doi:10.3390/plants13101321)
Supplement: Supplementary file 1 [file plants-13-01321-s001.zip › 1.Supplementary Figure.pdf]

Supplementary figure

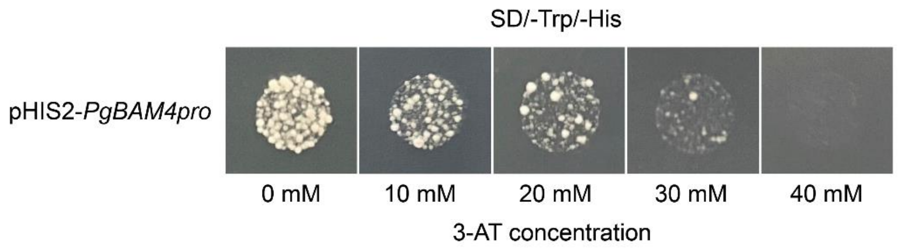

Figure S1. Screening of the optimal concentration of 3-AT used for yeast one hybrid assay. The yeast strain Y187 transformed with the pHIS2-*PgBAM4pro* vectors were grown on -Trp/-His medium containing different concentrations of 3-AT.
